# Exendin-4 Prevents Vascular Smooth Muscle Cell Proliferation and Migration by Angiotensin II via the Inhibition of ERK1/2 and JNK Signaling Pathways

**DOI:** 10.1371/journal.pone.0137960

**Published:** 2015-09-17

**Authors:** Kosuke Nagayama, Yoji Kyotani, Jing Zhao, Satoyasu Ito, Kentaro Ozawa, Francesco A. Bolstad, Masanori Yoshizumi

**Affiliations:** 1 Department of Pharmacology, Nara Medical University School of Medicine, Kashihara, Nara, Japan; 2 Department of Clinical English, Nara Medical University School of Medicine, Kashihara, Nara, Japan; National Center for Scientific Research Demokritos, GREECE

## Abstract

Angiotensin II (Ang II) is a main pathophysiological culprit peptide for hypertension and atherosclerosis by causing vascular smooth muscle cell (VSMC) proliferation and migration. Exendin-4, a glucagon-like peptide-1 (GLP-1) receptor agonist, is currently used for the treatment of type-2 diabetes, and is believed to have beneficial effects for cardiovascular diseases. However, the vascular protective mechanisms of GLP-1 receptor agonists remain largely unexplained. In the present study, we examined the effect of exendin-4 on Ang II-induced proliferation and migration of cultured rat aortic smooth muscle cells (RASMC). The major findings of the present study are as follows: (1) Ang II caused a phenotypic switch of RASMC from contractile type to synthetic proliferative type cells; (2) Ang II caused concentration-dependent RASMC proliferation, which was significantly inhibited by the pretreatment with exendin-4; (3) Ang II caused concentration-dependent RASMC migration, which was effectively inhibited by the pretreatment with exendin-4; (4) exendin-4 inhibited Ang II-induced phosphorylation of ERK1/2 and JNK in a pre-incubation time-dependent manner; and (5) U0126 (an ERK1/2 kinase inhibitor) and SP600125 (a JNK inhibitor) also inhibited both RASMC proliferation and migration induced by Ang II stimulation. These results suggest that exendin-4 prevented Ang II-induced VSMC proliferation and migration through the inhibition of ERK1/2 and JNK phosphorylation caused by Ang II stimulation. This indicates that GLP-1 receptor agonists should be considered for use in the treatment of cardiovascular diseases in addition to their current use in the treatment of diabetes mellitus.

## Introduction

The pathogenesis of atherosclerosis is multifactorial, including vasoconstriction, thromboembolism, and vascular smooth muscle cell (VSMC) proliferation and migration [1]. Among various circulatory factors, angiotensin II (Ang II) is a contributing pathophysiological peptide for hypertension and atherosclerosis [2]. Ang II has various actions on VSMC; modulating vasomotor tone, regulating cell growth and apoptosis, influencing cell migration and extracellular matrix deposition, causing inflammatory reactions, and stimulating production of other growth factors and vasoconstrictors [3]. Earlier studies have suggested that the Ang II-induced cellular responses are attributable, in part, to the phosphorylation of intracellular signaling molecules including the mitogen-activated protein (MAP) kinase family, which includes extracellular signal-regulated kinase (ERK)1/2 and c-Jun N-terminal kinase (JNK) [4]. Ang II has been reported as phosphorylating ERK1/2 and JNK in VSMC, members of the MAP kinase family [5] [6].

Glucagon-like peptide 1(GLP-1) is a 30-amino acid peptide hormone that is secreted from gut endocrine cells and is stimulated by nutrient ingestion inducing glucose-dependent insulin secretion through the activation of G-protein-coupled GLP-1 receptors on pancreatic islet cells [7] [8] [9]. Activation of GLP-1 receptors induces the generation of cAMP through the action of adenylate cyclase, then cAMP signaling cascades are amplified and diversified through the activation of various downstream effectors, including protein kinase A, Protein kinase C and phosphatidylinositol-3 kinase [10]. However, because GLP-1 is rapidly metabolized by its degrading enzyme dipeptidyl peptidase-4 (DPP-4), the half-life of intact GLP-1 is very short (less than 2 min) [11]. A GLP-1 receptor agonist, exendin-4 was originally isolated from the salivary gland of Glia monster lizards. It is resistant to cleavage by DPP-4 and therefore shows long lasting activity, making it a prime candidate for the treatment of type 2 diabetes [12] [13]. In addition to its blood glucose lowering effect through the stimulation of insulin secretion from the pancreas, GLP-1 has pleiotropic effects on various other organs and tissues [14]. For example, GLP-1 exerts a neuroprotective effect on the brain, a glycogen storage effect on the liver and a natriuretic effect on the kidneys [14]. Recently, accumulating evidence suggests the cardiovascular effects of GLP-1 in modulating vascular tone [15] and blood pressure [16]. GLP-1 also has cardioprotective effects, such as inhibiting hypertensive heart failure in rats [17] and improving the survival rate of myocardial infarction in mice [18]. In addition, GLP-1 analogue also upregulates nitric oxide production and exerts an anti-inflammatory effect in vascular endothelial cells [19]. It has also been reported that exendin-4 reduced intimal thickening after vascular injury through the inhibition of VSMC proliferation [20]. However, the precise beneficial effects of GLP-1 on the cardiovascular system are largely unknown and its mechanisms still remain to be explained.

In the present study, we examined the effect of exendin-4 on Ang II-induced proliferation and migration of cultured rat aortic smooth muscle cells (RASMC) to explore the vascular protective effect of GLP-1 receptor agonist. The effect of exendin-4 on changes in intracellular signaling by Ang II was also examined to provide a possible mechanism by which exendin-4 may be used as a pharmacotherapeutic agent for the prevention of hypertension and atherosclerosis independent of its blood glucose lowering effect.

## Materials and Methods

### Cell culture

This study was carried out in strict accordance with the recommendations in the Guide for the Care and Use of Laboratory Animals of the National Institutes of Health. The protocol was approved by Nara Medical University’s Committee on the Ethics of Animal Experiments (Permit Number: 11011). All surgery was performed under the inhalation of isoflurane anesthesia, and all efforts were made to minimize suffering.

RASMC were isolated from the thoracic aorta of 8-week-old male Sprague–Dawley rats by enzymatic digestion, as previously described [21]. Cells were grown in Dulbecco’s modified Eagle’s medium with 4.5g/L glucose (DMEM, Sigma-Aldrich, St Louis, MO) supplemented with 10% fetal bovine serum (FBS, HyClone, Logan, UT), penicillin (100 U/mL, Invitrogen, Carlsbad, CA), and streptomycin (100 μg/mL, Invitrogen) at 37°C under 5% CO_2_ in a humidified incubator. After reaching confluence in three 35-mm dishes, the cells were harvested by brief trypsinization and grown in T-75 flasks (Iwaki, Osaka, Japan) (passage 1). RASMC were used for experiments between the third and sixth passages. When the cell confluency in culture was estimated to be 50% to over 90%, the medium was replaced with unsupplemented DMEM. The cells were further cultured for 48 h and then subjected to each experiment. In some experiments, RASMC were pre-incubated with exendin-4 for 5 min to 1h and MAP kinase inhibitors (U0126, an ERK1/2 kinase inhibitor, or SP600125, a JNK inhibitor) for 1 h prior to stimulation with Ang II.

### WST-8 assay and cell counting with Burker-Turk for measurement of cell proliferation

A cell counting assay was performed essentially according to the manufacturer’s protocol as described below [22] [23]. In brief, after each experiment, WST-8 reagent [2-(2-methoxy-4-nitrophenyl)-3-(4-nitrophenyl)-5-(2,4-disulfophenyl)-2H-tetrazolium, monosodium salt] from Cell Counting Kit-8 (Dojindo, Mashikimachi, Japan) was added to each cell medium (finally 10% (v/v)). Then, cells were incubated at 37°C for another 0.5 to 1.5 h. The formation of formazan was determined photometrically at 450 nm (reference wave length at 650 nm) with a Sunrise™ microplate reader (Tecan, Männedorf, Switzerland). In Addition, direct cell number counting was performed with the cell counting plate. After each experiment, RASMC were trypsinized for 5 min at 37°C and transferred into 300 μl DMEM for cell number counting using a cell counting plate (Burker-Turk).

### Wound healing assay for measurement of cell migration

We performed a wound healing assay to evaluate cell migration, especially transverse displacement. After culturing in serum-free DMEM for 24 h, the cells were incubated in the absence or presence of exendin-4 or MAP kinase inhibitors for 1 h. Then, the cells were treated with Ang II, wounded using a yellow pipette tip, and cultured for 24 h. Later, the wounded cultures were stained with Diff-Quick (Sysmex, Kobe, Japan) and photographed. Cells that had migrated from the rim of the dishes were counted in 10 microscope fields per dish.

### Western blot analysis

For western blot analysis, RASMC were lysed immediately after each experiment and lysate proteins were collected in the manner described earlier [24]. The cellular proteins were denatured and subjected to SDS-polyacrylamide gel electrophoresis. The proteins were transferred onto PVDF membranes (0.45 μm pore size), according to the method described previously [21]. The membranes for each blot were blocked by 5% skim milk (Nacalai Tesque, Kyoto, Japan) in tris-buffered saline containing 0.1% Tween-20 (TBS-T) for 1 h at room temperature. The blots were incubated overnight at 4°C with primary antibody against SM22α (MBL, Nagoya, Japan), phospho-Yes-associated protein 1 (Yap1), ERK1/2, phospho-ERK1/2, JNK, or phospho-JNK (Cell Signaling Technology, Danvers, MA, USA) and then, with secondary antibody conjugated with POD (Santa Cruz Biotechnology, Inc., Santa Cruz, CA, USA) at room temperature for 1 h. Immunoreactive bands were visualized using enhanced chemiluminescence (Amersham, Buckinghamshire, UK) and quantified by densitometry. The values of phospho-MAP kinase have been normalized to total MAP kinase measurements and then expressed as the ratio of normalized values to protein in the control group as 1 (n = 4 per group). Band intensities were quantified by densitometry of the immunoblots using NIH Image J software.

### Materials

Materials were purchased from Wako (Kyoto) or Nacalai Tesque (Kyoto) unless stated otherwise. Exendin-4 was procured from Sigma-Aldrich (St Louis, MO). The antibodies used for the western blot analyses were purchased as follows: anti SM22α, anti phospho-Yes-associated protein 1 (Yap1), anti-phospho-ERK1/2 antibody and anti-phospho-SAPK/JNK antibodies were purchased from Cell Signaling Technology, while ECL and ECL plus systems were purchased from GE Healthcare. Collagen I was purchased from Nippon Meat Packers, Inc. (Osaka). All chemical compounds were dissolved in dimethyl sulfoxide (DMSO) at final concentration of less than 1% except where specially noted.

### Statistical analysis

All experimental values were expressed as mean ± standard error of the mean. Non-parametric ANOVA analysis with Kruskal-Wallis and Dunn’s correction was used to determine significant differences in multiple comparisons. A *P* value <0.05 was considered to be significant.

## Results

### Ang II caused a phenotypic switch of RASMC from contractile type to synthetic proliferative type cells

**Fig 1:**
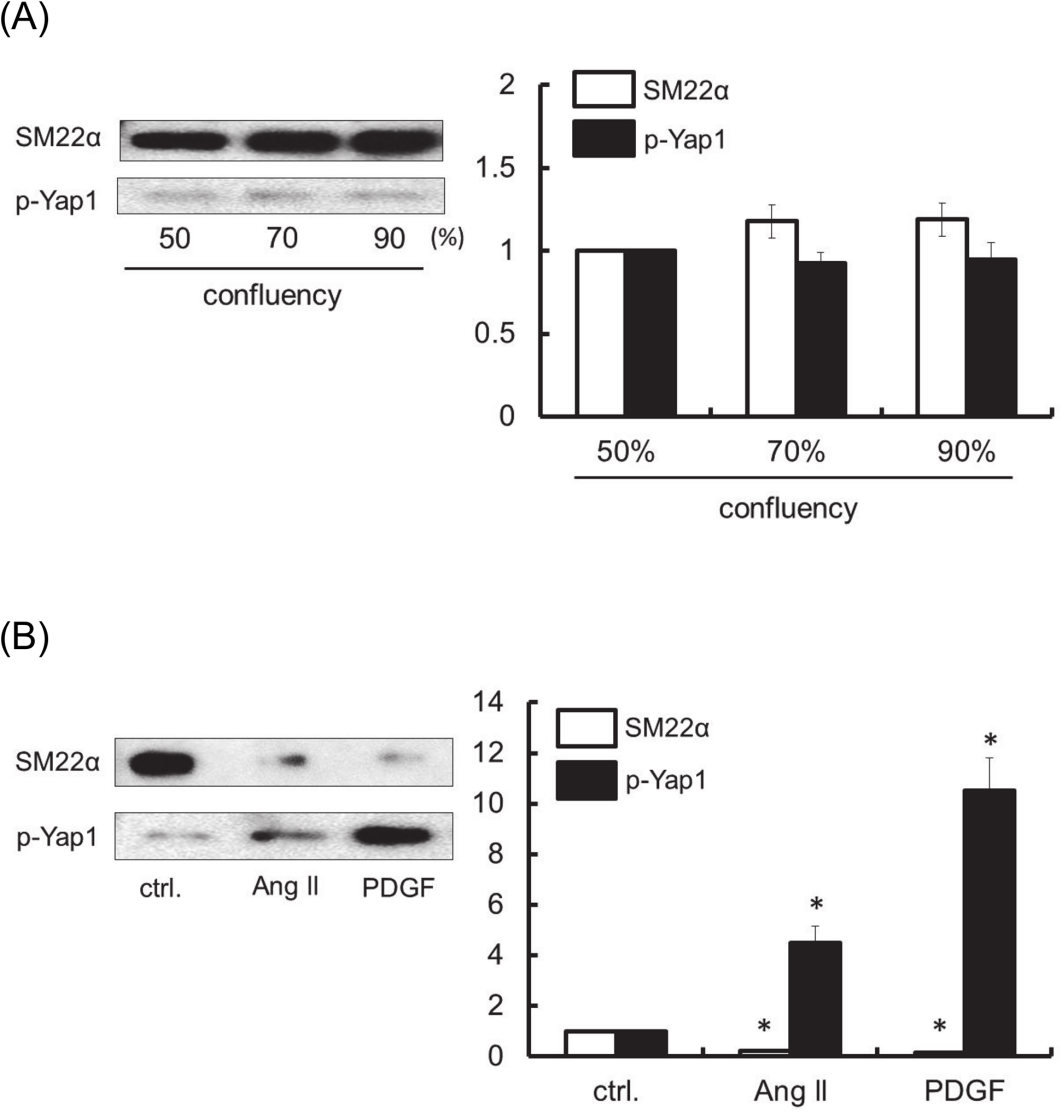
Influence of the cell confluency (A) and the effect of Ang II (B) on the change in phenotypic characteristics of RASMC. The phenotypic character of RASMC were determined by western blot analysis using SM22α antibody, a marker for contractile phenotype and phospho-Yap1 antibody, a marker for synthetic proliferative phenotype. Growth-arrested RASMC with 50%, 70% and 90% confluency were analysed (A). The growth-arrested cells were exposed to Ang II (100 nM) or PDGF-BB (10 ng/mL) and then incubated for 24 h (B). SM22α and phospho-Yap1 were evaluated by western blotting assay as described under Materials and Methods. Densitometric analysis of each value was normalized by arbitrarily setting the densities of the negative control (ctrl.) (50% confluent cells or without agonist stimulation) to 1. Each value represents the mean ± standard error (S.E.) (n = 4). The asterisks represent significant differences compared with the negative control value (*P < 0.05).

We firstly examined the phenotypic character of RASMC by western blot analysis using SM22α antibody, a marker for contractile phenotype, and phospho-Yap1 antibody, a marker for synthetic proliferative phenotype [25]. As shown in Fig 1A, growth-arrested RASMC showed primarily SM22α positive contractile phenotype and no significant difference was observed among 50%, 70% and 90% confluency samples of RASMC. However, as well as platelet-derived growth factor (PDGF) BB, Ang II treatment for 24 h caused a significant phenotypic switch from contractile type cells to synthetic proliferative type cells (Fig 1B).

### Concentration-response for the Ang II-induced RASMC proliferation and its inhibition by exendin-4

**Fig 2:**
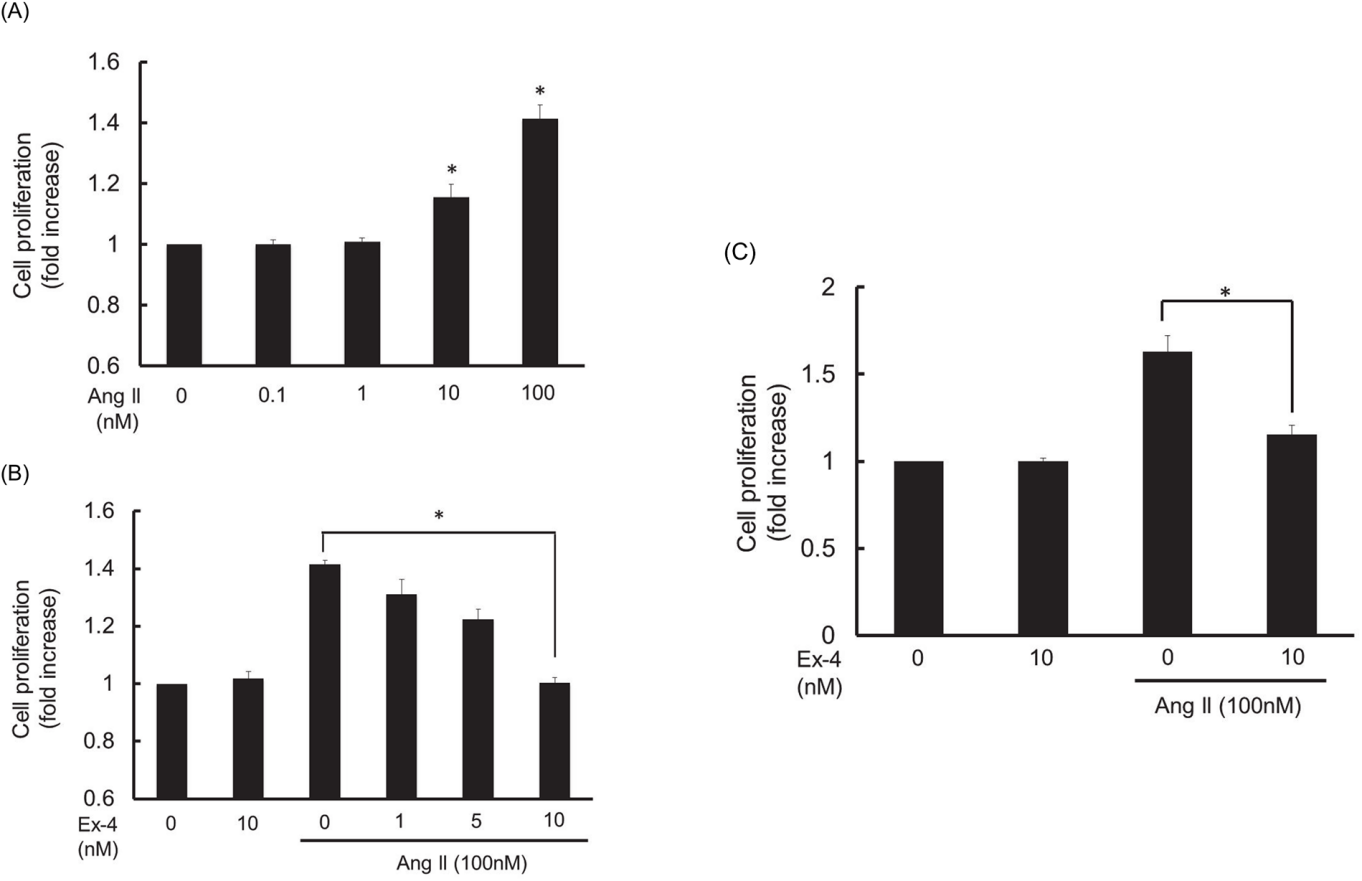
Concentration response for the effect of Ang II on RASMC proliferation (A) and the effect of exendin-4 (Ex-4) on 100 nM of Ang II-induced RASMC proliferation (B). The growth-arrested cells were exposed to various concentrations of Ang II (from 0.1 nM to 100 nM) and then incubated for 24 h. Various concentrations of exendin-4 (from 1 nM to 10 nM) were added to the medium 1 h prior to Ang II stimulation. Cell proliferation was evaluated by WST-8 assay as described under Materials and Methods. Colorimetric analysis of each value was normalized by arbitrarily setting the absorbance value of the control cells to 1. Ang II-induced RASMC proliferation and its inhibition by exendin-4 were also measured by direct cell number counting with a Burker-Turk cell counting plate as described under Materials and Methods (C). Data were corrected for replication of unstimulated RASMC and the index of cell number indicated as the x fold increase over the baseline value of RASMC. Each value represents the mean ± standard error (S.E.) (n = 4). The asterisks represent significant differences compared with the negative or positive control value (*P < 0.05).

To evaluate the relative magnitude of Ang II-induced VSMC proliferation, growth-arrested RASMC were exposed to various concentrations of Ang II. Ang II-induced cell proliferation was determined as described under Materials and Methods. As shown in Fig 2A, Ang II caused concentration-dependent RASMC proliferation and significant increase in cell numbers was observed at levels above 10 nM of Ang II. We next examined the effect of exendin-4 on 100 nM of Ang II-induced RASMC proliferation. Exendin-4 exhibited a concentration-dependent inhibition on Ang II-induced RASMC proliferation and a significant difference in the proliferation of RASMC was observed at 10 nM (Fig 2B). Ang II induced an increase in cell numbers and its inhibition by exendin-4 was also confirmed by direct cell counting assay using a Burker-Turk cell counting plate (Fig 2C).

### Concentration-response for the Ang II-induced RASMC migration and its inhibition by exendin-4

**Fig 3:**
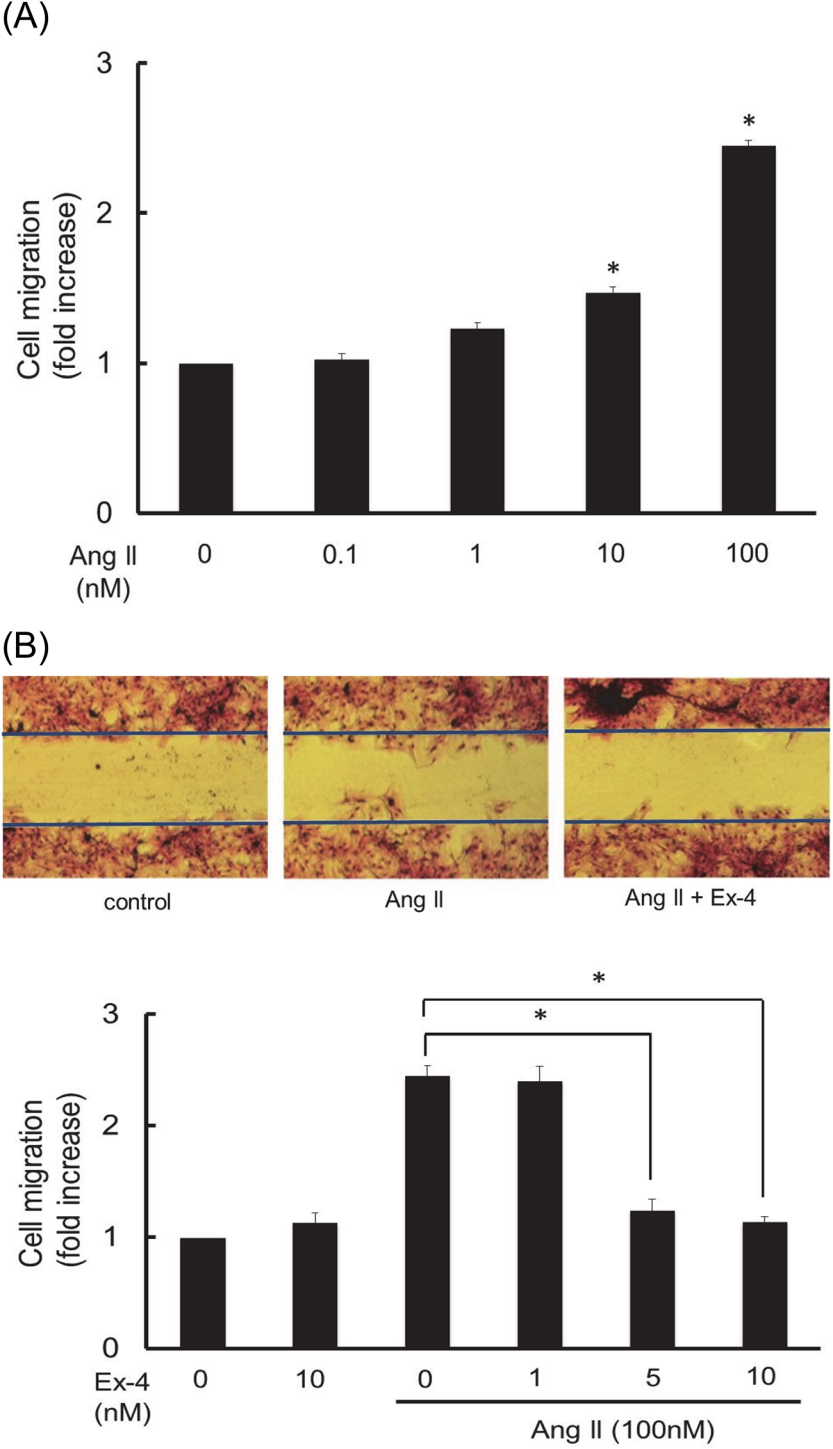
Concentration response for the effect of Ang II on RASMC migration (A) and the effect of exendin-4 (Ex-4) on 100 nM of Ang II-induced RASMC migration (B). The growth-arrested cells were exposed to various concentrations of Ang II (from 0.1 nM to 100 nM) and then incubated for 24 h. Various concentrations of exendin-4 (from 1 nM to 10 nM) were added to the medium 1 h prior to Ang II stimulation. Cell migration was evaluated through a wound healing assay as described under Materials and Methods. Migrated cell counting analysis of each value was normalized by arbitrarily setting the cell numbers of the control cells to 1. Each value represents the mean ± standard error (S.E.) (n = 4). The asterisks represent significant differences compared with the negative or positive control value (*P < 0.05).

Next, to examine the concentration-response for the Ang II-induced VSMC migration, growth-arrested RASMC were exposed to various concentrations of Ang II. Ang II-induced cell migration was measured as described under Materials and Methods. As shown in Fig 3A, Ang II induced a concentration-dependent RASMC migration and a significant increase in cell migration was observed at levels above 10 nM of Ang II. Next we examined the effect of exendin-4 on 100 nM of Ang II-induced RASMC migration. As shown in Fig 3B, Exendin-4 showed concentration-dependent inhibition on Ang II-induced RASMC migration and a significant difference was observed at 5 nM concentration of exendin-4 compared to the Ang II-induced positive control. These results suggest that exendin-4 is a potent inhibitor for Ang II-induced VSMC proliferation and migration.

### Effect of exendin-4 on Ang II-induced activation of ERK1/2 and JNK in RASMC

**Fig 4:**
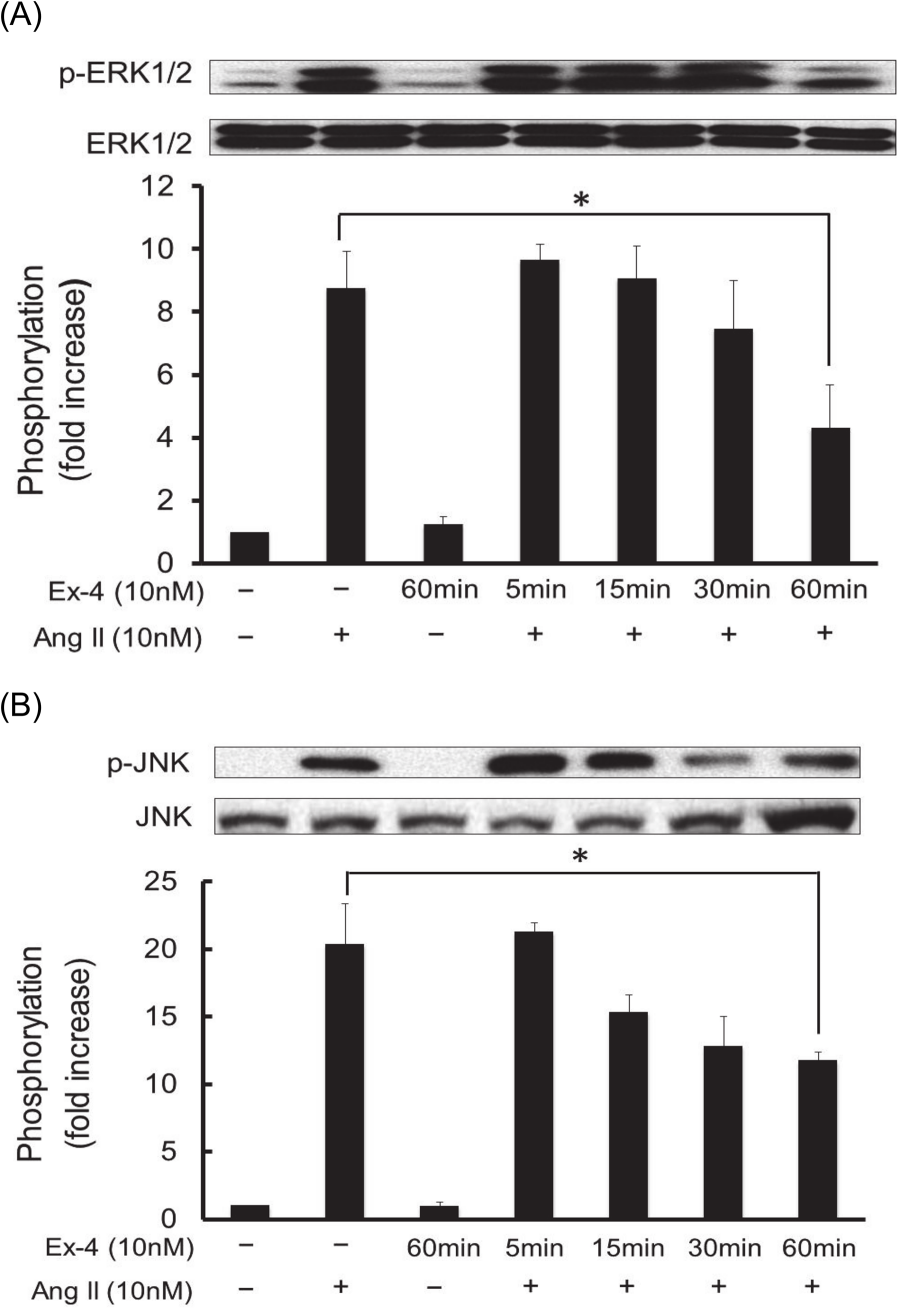
Ang II-iduced phosphorylation of ERK1/2 (A) and JNK (B) and pre-incubation time-dependent inhibition by exendin-4 in RASMC. The growth-arrested cells were exposed to 10 nM of Ang II for 5 min. Exendin-4 (Ex-4) was added to the medium 5 min to 1 h prior to Ang II stimulation. ERK1/2 and JNK phosphorylation were evaluated by western blotting assay as described under Materials and Methods. Densitometric analysis of each value was normalized by arbitrarily setting the densities of the negative control (without Ang II stimulation) to 1. Each value represents the mean ± standard error (S.E.) (n = 4). The asterisks represent significant differences compared with the positive control value (*P < 0.05).

In order to clarify the intracellular signaling mechanisms associated with exendin-4 inhibition of Ang II-induced RASMC proliferation and migration, the following experiments were undertaken. The effects of Ang II on the phosphorylation of ERK1/2 and JNK were assessed by western blot analysis as described under Materials and Methods. As shown in Fig 4A and 4B, ERK1/2 and JNK were rapidly phosphorylated by Ang II at 10 nM with 5 min stimulation in RASMC. In addition, exendin-4 significantly inhibited Ang II-induced ERK1/2 and JNK phosphorylation in a pre-incubation time-dependent fashion from 5 min to 60 min (Fig 4A and 4B). These findings suggest that exendin-4 inhibited Ang II-induced RASMC proliferation and migration through the inhibition of ERK1/2 and JNK activation by Ang II stimulation.

### Inhibitory effect of U0126 and SP600125 on Ang II-induced RASMC proliferation and migration

**Fig 5:**
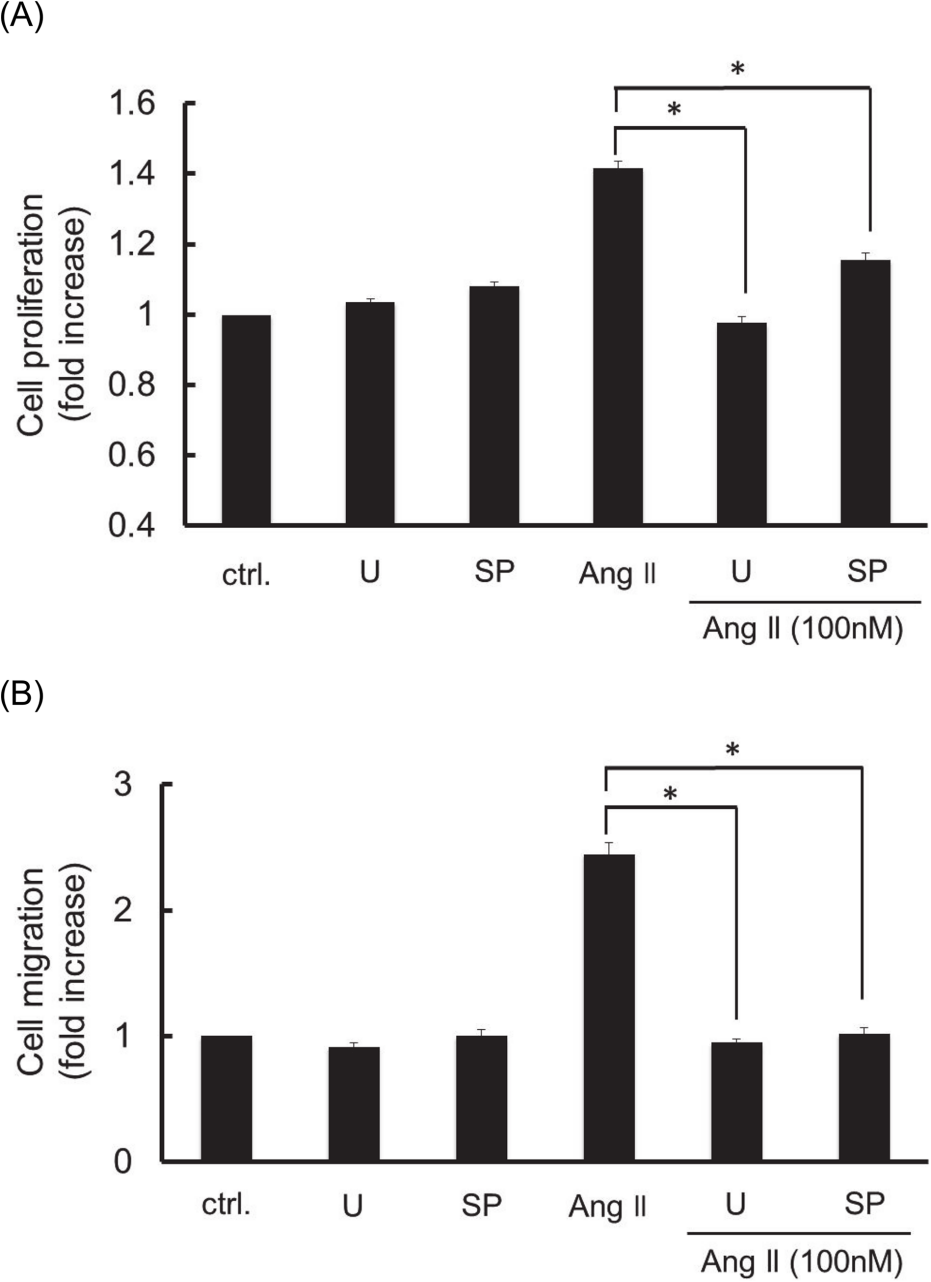
Effects of U0126 (an ERK1/2 kinase inhibitor) and SP600125 (a JNK inhibitor) on the cell proliferation (A) and migration (B) induced by Ang II in RASMC. The cells were pre-incubated with or without U0126 (30 μM), and SP600125 (3 μM) for 1h prior to exposing to Ang II (100 nM) for 24 h. U0126, and SP600125 are abbreviated as U, and SP. Cell proliferation was evaluated by WST-8 assay and cell migration was evaluated by wound healing assay, respectively. Colorimetric analysis and migrated cell counting analysis of each value was normalized by arbitrarily setting the value of the control cells (ctrl.) to 1. Each value represents the mean ± standard error (S.E.) (n = 6). The asterisks represent significant differences compared with the positive control value (*P < 0.05).

To confirm the possible involvement of ERK1/2 and JNK on Ang II-induced RASMC proliferation and migration, we examined the effects of U0126 (an ERK1/2 kinase inhibitor) and SP600125 (a JNK inhibitor) on Ang II-induced RASMC proliferation and migration. As shown in Fig 5A, both U0126 and SP600125 significantly inhibited Ang II-induced RASMC proliferation. U0126 and SP600125 also showed almost complete inhibition of Ang II-induced RASMC migration (Fig 5B). These findings suggest that ERK1/2 and JNK play important roles in Ang II-induced VSMC proliferation and migration.

## Discussion

The major findings of the present study are as follows: (1) Ang II caused a phenotypic switch of RASMC from contractile type to synthetic type cells; (2) Ang II caused concentration-dependent RASMC proliferation, which was significantly inhibited by pre-treatment with exendin-4; (3) Ang II caused concentration-dependent RASMC migration, which was significantly inhibited by pre-treatment with exendin-4; (4) exendin-4 inhibited Ang II-induced phosphorylation of ERK1/2 and JNK in a pre-incubation time-dependent manner; and (5) as well as exendin-4, ERK1/2 kinase and JNK inhibitors also inhibited both RASMC proliferation and migration induced by Ang II stimulation.

Ang II is known to cause hypertension through type 1 receptor mediated vasoconstriction and stimulation of aldosterone secretion from the adrenal cortex, which are associated with vascular remodeling, inflammation and oxidative stresses [3]. The main causes of vascular remodeling are excessive proliferation and migration of VSMC, which are characteristic features of the pathogenesis of atherosclerosis [2]. As shown in Fig 2A, Ang II caused RASMC proliferation in a concentration-dependent manner. The magnitude of Ang II-induced RASMC proliferation was not remarkable at concentrations under 10 nM; however, a significant difference was observed at concentrations over than 10 nM of Ang II. Exendin-4 at 10 nM significantly inhibited Ang II-induced RASMC proliferation (Fig 2B). Ang II-induced an increase in cell numbers and its inhibition by exendin-4 was also confirmed by direct cell counting (Fig 2C). RASMC migration, which is another characteristic feature of vascular remodeling, was also examined. As shown in Fig 3A and 3B, Ang II at a concentration of 100 nM caused significant RASMC migration, which was inhibited by pre-treatment with exendin-4 at 10 nM. These findings suggest that exendin-4 may have a preventative effect on the progression of atherosclerosis through the inhibition of VSMC proliferation and migration. Other researchers have also reported that GLP-1 prevented the development of atherosclerosis in apolipoprotein E knockout mice [26]. In addition, it was also reported that exendin-4 reduced the atherosclerotic lesion of apolipoprotein E knockout mice through the inhibition of monocyte adhesion to endothelial cells [27]. Viewed in conjunction with these findings, our findings suggest that exendin-4 may exert a vascular protective effect through the inhibition of VSMC proliferation and migration. However, further in vivo studies are needed to clarify the beneficial effect of exendin-4 on the cardiovascular system using animal models, such as spontaneously hypertensive rats or DOCA/salt rats.

Previously, we reported that Ang II stimulated RASMC migration through the activation of intracellular ERK1/2 and JNK [28] [29]. It was also reported that Ang II caused VSMC proliferation through the mechanisms of ERK1/2 and JNK signaling pathways [30]. As shown in Fig 4A and 4B, Ang II rapidly stimulated ERK1/2 and JNK phosphorylation, both of which were inhibited by exendin-4 in a pre-incubation time-dependent manner in RASMC. In addition, both U0126 (an ERK1/2 kinase inhibitor) and SP600125 (a JNK inhibitor) inhibited Ang II-induced RASMC proliferation and migration (Fig 5A and 5B). These findings suggest that exendin-4 inhibits Ang II-induced RASMC proliferation and migration, in part through the inhibition of ERK1/2 and JNK signaling pathways. Investigation of GLP-1 receptor-mediated intracellular signaling shows that GLP-1 receptor activation causes an increase in cAMP, which results in the potentiation of insulin secretion from pancreatic islet β-cells [31]. Ras and MAP kinase-dependent signaling pathways have also been found to lead to an increase in insulin gene expression and insulin content in pancreatic β-cells [32]. Although GLP-1 receptor agonists-induced intracellular signaling mechanisms are well investigated in pancreatic islet β-cells, little is known about the GLP-1 receptor mediated signaling in VSMC. In the present study, we found that exendin-4 inhibited Ang II-induced ERK1/2 and JNK phosphorylation in RASMC (Fig 4A and 4B). Consistent with our findings, Zhao et al. reported that exendin-4 alleviates Ang II-induced senescence in VSMC by inhibiting Rac1 activation through the cAMP/protein kinase A-dependent pathway [33]. However, in their study, Zhao et al. concluded that the inhibitory effect of exendin-4 is attributable to its inhibition on the generation of oxidative stress through NAD(P)H oxidase activation induced by Ang II stimulation. Hirata et al. also reported that exendin-4 inhibited platelet-derived growth factor-induced VSMC proliferation through the inhibition of the cAMP/ protein kinase A-dependent pathway without referring to the MAP kinase signaling pathway [34]. Taken together, we show for the first time that exendin-4 inhibits VSMC proliferation and migration, at least in part through the inhibition of ERK1/2 and JNK phosphorylation induced by Ang II stimulation. The importance of ERK1/2 and JNK activation was confirmed by the finding that both the ERK1/2 kinase inhibitor and JNK inhibitor effectively inhibited Ang II-induced RASMC proliferation and migration (Fig 5A and 5B).

In conclusion, exendin-4 inhibited RASMC proliferation and migration induced by Ang II stimulation. ERK1/2 and JNK in RASMC were phosphorylated by Ang II; however, these activations were inhibited by pretreatment with exendin-4. Similar to exendin-4, pharmacological inhibition of ERK1/2 and JNK activation by Ang II suppressed Ang II-induced RASMC proliferation and migration. The mechanism for progression of atherosclerosis will need to be clarified by further study on the fate of VSMC influenced by circulatory factors including Ang II. We hope our present findings shed light on the mechanisms of the cardioprotective effect of GLP-1 receptor agonists beyond its blood glucose lowering effect.

## Supporting Information

S1 Text ARRIVE checklist. (DOCX)
