# Supplementary material for: Exendin-4 Prevents Vascular Smooth Muscle Cell Proliferation and Migration by Angiotensin II via the Inhibition of ERK1/2 and JNK Signaling Pathways
Source: PLoS One. 2015 Sep 17;10(9):e0137960. doi: 10.1371/journal.pone.0137960 (PMC4574935; doi:10.1371/journal.pone.0137960)
Supplement: S1 Text — (DOCX) [file pone.0137960.s001.docx]

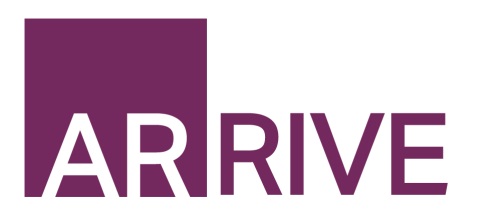


The ARRIVE Guidelines Checklist

Animal Research: Reporting In Vivo Experiments

Carol Kilkenny^1^, William J Browne^2^, Innes C Cuthill^3^, Michael Emerson^4^ and Douglas G Altman^5^

*^1^The National Centre for the Replacement, Refinement and Reduction of Animals in Research, London, UK, ^2^School of Veterinary Science, University of Bristol, Bristol, UK, ^3^School of Biological Sciences, University of Bristol, Bristol, UK, ^4^National Heart and Lung Institute, Imperial College London, UK, ^5^Centre for Statistics in Medicine, University of Oxford, Oxford, UK.*

|  | ITEM | RECOMMENDATION | Section/ Paragraph |
| --- | --- | --- | --- |
| Title | 1 | Provide as accurate and concise a description of the content of the article as possible. | To examine the effect of exendin-4 on cultured rat aortic smooth muscle cell proliferation and migration induced by angiotensin II (Ang II) stimulation. |
| Abstract | 2 | Provide an accurate summary of the background, research objectives, including details of the species or strain of animal used, key methods, principal findings and conclusions of the study. | To investigate the mechanism of the progression of atherosclerosis, we examined the effects of exendin-4 and mitogen-activated protein kinase inhibitors on Ang II-induced rat aortic smooth muscle cell proliferation and migration. Two male Sprague–Dawley rats were used to obtain aortic smooth muscle cells for culture. Exendin-4 inhibited cultured rat aortic smooth muscle cell proliferation and migration induced by Ang II stimulation. MAP kinase inhibitors also inhibited cultured rat aortic smooth muscle cell proliferation and migration by Ang II stimulation. Exendin-4 may be an alternative candidate for prevention of the progression of atherosclerosis independent of its blood glucose lowering effect in humans. |
| INTRODUCTION | | |  |
| Background | 3 | a. Include sufficient scientific background (including relevant references to previous work) to understand the motivation and context for the study, and explain the experimental approach and rationale.  b. Explain how and why the animal species and model being used can address the scientific objectives and, where appropriate, the study’s relevance to human biology. | The pathogenesis of atherosclerosis is multifactorial, including vasoconstriction, thromboembolism, and vascular smooth muscle cells (VSMC) proliferation and migration [1]. Among various neurohumoral factors, angiotensin II (Ang II) is a key effector peptide for hypertension and atherosclerosis [2]. A GLP-1 receptor agonist, exendin-4 was originally isolated from the salivary gland of Glia monster lizards. It is resistant to cleavage by DPP-4 and therefore shows long lasting activity for the treatment of type 2 diabetes [3] [4]. However, the precise beneficial effects of GLP-1 on the cardiovascular system are largely unknown and its mechanisms still remained to be elucidated. In the present study, we examined the effects of exendin-4 and mitogen-activated protein kinase inhibitors on Ang II-induced rat aortic smooth muscle cell proliferation and migration. Two male Sprague–Dawley rats were used to obtain aortic smooth muscle cells for culture. Exendin-4 inhibited cultured rat aortic smooth muscle cell proliferation and migration induced by Ang II stimulation. Our findings may be applied to the treatment of hypertension and atherosclerosis in humans. |
| Objectives | 4 | Clearly describe the primary and any secondary objectives of the study, or specific hypotheses being tested. | The primary object of the present study is to examine the effect of exendin-4 on Ang II-induced proliferation and migration of cultured rat aortic smooth muscle cells. The changes in intracellular signalling by Ang II were also examined. |
| METHODS | | |  |
| Ethical statement | 5 | Indicate the nature of the ethical review permissions, relevant licences (e.g. Animal [Scientific Procedures] Act 1986), and national or institutional guidelines for the care and use of animals, that cover the research. | The study design was approved by Nara Medical University’s ethics review board under the guidelines for the use of laboratory animals of Nara Medical University (No. 11011) and this study was conducted in accordance with the guide for the Care and Use of Laboratory Animals as adopted and promulgated by the United States National Institute of Health. |
| Study design | 6 | For each experiment, give brief details of the study design including:  a. The number of experimental and control groups.  b. Any steps taken to minimise the effects of subjective bias when allocating animals to treatment (e.g. randomisation procedure) and when assessing results (e.g. if done, describe who was blinded and when).  c. The experimental unit (e.g. a single animal, group or cage of animals).  A time-line diagram or flow chart can be useful to illustrate how complex study designs were carried out. | N/A |
| Experimental procedures | 7 | For each experiment and each experimental group, including controls, provide precise details of all procedures carried out. For example:  a. How (e.g. drug formulation and dose, site and route of administration, anaesthesia and analgesia used [including monitoring], surgical procedure, method of euthanasia). Provide details of any specialist equipment used, including supplier(s).  b. When (e.g. time of day).  c. Where (e.g. home cage, laboratory, water maze).  d. Why (e.g. rationale for choice of specific anaesthetic, route of administration, drug dose used). | N/A |
| Experimental animals | 8 | a. Provide details of the animals used, including species, strain, sex, developmental stage (e.g. mean or median age plus age range) and weight (e.g. mean or median weight plus weight range).  b. Provide further relevant information such as the source of animals, international strain nomenclature, genetic modification status (e.g. knock-out or transgenic), genotype, health/immune status, drug or test naïve, previous procedures, etc. | Two male Sprague–Dawley rats at 8 weeks of age (Kiwa Animal, Nara, Japan) were sacrificed with intravenous infusion of pentobarbital. The thoracic aortae were excised and were subjected to smooth muscle cell harvesting by an explant method [5]. Vendor health reports indicated that the rats were free of known viral, bacterial and parasitic pathogens. |

The ARRIVE guidelines. Originally published in *PLoS Biology*, June 2010^1^

| Housing and husbandry | 9 | | Provide details of:  a. Housing (type of facility e.g. specific pathogen free [SPF]; type of cage or housing; bedding material; number of cage companions; tank shape and material etc. for fish).  b. Husbandry conditions (e.g. breeding programme, light/dark cycle, temperature, quality of water etc for fish, type of food, access to food and water, environmental enrichment).  c. Welfare-related assessments and interventions that were carried out prior to, during, or after the experiment. | N/A |  |
| --- | --- | --- | --- | --- | --- |
| Sample size | 10 | | a. Specify the total number of animals used in each experiment, and the number of animals in each experimental group.  b. Explain how the number of animals was arrived at. Provide details of any sample size calculation used.  c. Indicate the number of independent replications of each experiment, if relevant. | N/A |  |
| Allocating animals to experimental groups | 11 | | a. Give full details of how animals were allocated to experimental groups, including randomisation or matching if done.  b. Describe the order in which the animals in the different experimental groups were treated and assessed. | N/A |  |
| Experimental outcomes | 12 | | Clearly define the primary and secondary experimental outcomes assessed (e.g. cell death, molecular markers, behavioural changes). | Exendin-4 inhibited cultured rat aortic smooth muscle cell proliferation and migration induced by Ang II stimulation. MAP kinases inhibitors also inhibited cultured rat aortic smooth muscle cell proliferation and migration by Ang II. |  |
| Statistical methods | 13 | | a. Provide details of the statistical methods used for each analysis.  b. Specify the unit of analysis for each dataset (e.g. single animal, group of animals, single neuron).  c. Describe any methods used to assess whether the data met the assumptions of the statistical approach. | The values of phospho-MAP kinase have been normalized to total MAP kinase measurements and then expressed as the ratio of normalized values to protein in the control group as 1 (n=3 per group). All experimental values were expressed as mean ± standard **error**. Analysis of variance along with a subsequent *t*-test was used to determine significant differences in multiple comparisons. A *P* value <0.05 was considered to be significant.  For each test, the experimental unit was cultured cells in a dish or well. |  |
| RESULTS | | | |  |  |
| Baseline data | 14 | | For each experimental group, report relevant characteristics and health status of animals (e.g. weight, microbiological status, and drug or test naïve) prior to treatment or testing. (This information can often be tabulated). | N/A |  |
| Numbers analysed | 15 | | 1. Report the number of animals in each group included in each analysis. Report absolute numbers (e.g. 10/20, not 50%^2^).   b. If any animals or data were not included in the analysis, explain why. | N/A |  |
| Outcomes and estimation | 16 | | Report the results for each analysis carried out, with a measure of precision (e.g. standard error or confidence interval). | N/A |  |
| Adverse events | 17 | | a. Give details of all important adverse events in each experimental group.  b. Describe any modifications to the experimental protocols made to reduce adverse events. | N/A |  |
| DISCUSSION | | | |  |  |
| Interpretation/ scientific implications | 18 | | a. Interpret the results, taking into account the study objectives and hypotheses, current theory and other relevant studies in the literature.  b. Comment on the study limitations including any potential sources of bias, any limitations of the animal model, and the imprecision associated with the results^2^.  c. Describe any implications of your experimental methods or findings for the replacement, refinement or reduction (the 3Rs) of the use of animals in research. | Exendin-4 inhibited rat aortic smooth muscle cell proliferation and migration induced by Ang II stimulation. ERK1/2 and JNK in rat aortic smooth muscle cells were activated by Ang II; however, the activation was inhibited by exendin-4. Similar to exendin-4, pharmacological inhibition of ERK1/2 and JNK suppressed Ang II-induced rat aortic smooth muscle cell proliferation and migration. It is expected that the mechanism of the progression of atherosclerosis will be clarified by further study of the fate of VSMC influenced by neurohumoral factors including Ang II.  To archive replacement, refinement or reduction (the 3Rs) of the use of animals in our research, vascular smooth muscle cell lines were used. |  |
| Generalisability/ translation | 19 | | Comment on whether, and how, the findings of this study are likely to translate to other species or systems, including any relevance to human biology. | Exendin-4 may be an alternative candidate for prevention of hypertension and atherosclerosis independent of its blood glucose lowering effect in humans. |  |
| Funding | 20 | List all funding sources (including grant number) and the role of the funder(s) in the study. | | The study was supported by a JSPS KAKENHI Grant, number 23590306 and 26460345, to MY. (http://www.e-rad.go.jp/index.html) | |


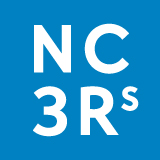


References:

1. Ross R (1999) Atherosclerosis--an inflammatory disease. N Engl J Med 340: 115-126.
2. Berk BC, Haendeler J, Sottile J (2000) Angiotensin II, atherosclerosis, and aortic aneurysms. J Clin Invest 105: 1525-1526.

3. Eng J, Kleinman WA, Singh L, Singh G, Raufman JP (1992) Isolation and characterization of exendin-4, an exendin-3 analogue, from Heloderma suspectum venom. Further evidence for an exendin receptor on dispersed acini from guinea pig pancreas. J Biol Chem 267: 7402-7405.

4. Kang JH, Chang SY, Jang HJ, Kim DB, Ryu GR, et al. (2009) Exendin-4 inhibits interleukin-1beta-induced iNOS expression at the protein level, but not at the transcriptional and posttranscriptional levels, in RINm5F beta-cells. J Endocrinol 202: 65-75.

5. Kyaw M, Yoshizumi M, Tsuchiya K, Kirima K, Tamaki T. (2001) Antioxidants inhibit JNK and p38 MAPK activation but not ERK 1/2 activation by angiotensin II in rat aortic smooth muscle cells. Hypertens Res. 24: 251-261.
